# Association of Tobacco Cessation With Cognitive Decline: A Multi-State Analysis

**DOI:** 10.7759/cureus.108784

**Published:** 2026-05-13

**Authors:** Mansi Joshi, Siya Patel, Danielle Fastring

**Affiliations:** 1 College of Medicine, William Carey University College of Osteopathic Medicine, Hattiesburg, USA; 2 Student Research, Preclinical Sciences, William Carey University College of Osteopathic Medicine, Hattiesburg, USA

**Keywords:** behavioral risk factor surveillance system (brfss) database, cognitive decline prevention, entorhinal cortex, neurology, preventative measures, tobacco cessation

## Abstract

Background: Mild cognitive decline and a faster reduction of entorhinal cortex volume are found to be more prevalent in individuals with a history of smoking, as shown in longitudinal and neuroimaging-based studies. Early screening of high-risk demographics may reduce cognitive decline progression.

Objective: The objective of this study is to understand the association between tobacco cessation and cognitive decline in the United States of America when adjusted for age, race, sex, education, and income, based on population-level survey data.

Methods: The data for this study were derived from the 2023 Behavioral Risk Factor Surveillance System (BRFSS), a population-based telephone survey that collects self-reported health information from non-institutionalized adults aged 18 years or older. Participants under 45 years old were excluded, as they were not administered questions from the BRFSS Cognitive Decline (CD) Module. Individuals from Florida, Maine, Rhode Island, Utah, Vermont, Wisconsin, Nevada, and Indiana participated in the CD module. Frequencies and percentages were computed for categorical demographic variables and the category of time since the individual stopped smoking. Bivariate analysis was used to determine whether the category of time since tobacco cessation and the demographic characteristics were independently associated with cognitive decline. Variables independently associated with CD (p≤0.10) were entered into a multivariable binary logistic model to determine the risk of CD for length of time category since smoking cessation when adjusted for age, race, sex, income, and education.

Results: This study identified a significant association between smoking cessation duration and cognitive decline (CD). Individuals who quit smoking 10 or more years prior had 3.648 times higher odds (95% CI 3.495, 3.807) of reporting CD compared to those who had never smoked regularly. Moreover, a general trend was observed, indicating greater CD with shorter durations since smoking cessation. The highest odds were observed among individuals who quit smoking between 1 month and less than 3 months prior, with an odds ratio of 5.466 (95% CI 5.197, 5.748) relative to the reference group. All analyses were adjusted for age, race, sex, income, and education.

Conclusion: This study concludes that individuals who have never smoked or quit at least 10 years ago are less likely to experience cognitive decline. A limitation is that the data is self-reported, which may introduce bias. The literature suggests smoking cessation is not linked to episodic memory loss, while smoking is associated with memory impairment. The effects of tobacco use on cognitive functioning may be reversed or halted with smoking cessation. Early intervention, including cognitive testing and smoking cessation counseling, may help reduce cognitive decline in smokers.

## Introduction

Cigarette smoking, even at low levels, is a well-established risk factor for vascular disease and stroke, which are both major contributors to dementia and cognitive decline. Smokers are at an elevated risk for a range of respiratory and cardiovascular diseases, all of which have been linked to cognitive impairment [1,2]. The chronic health conditions associated with smoking, such as hypertension and atherosclerosis, can negatively impact brain function, further heightening the risk of cognitive decline [3,4]. Despite this, the relationship between smoking cessation and cognitive decline remains somewhat ambiguous. While there is growing recognition of smoking as a modifiable risk factor for dementia in older adults, its full impact on cognitive decline and dementia is likely underestimated in many studies. This underestimation may be due to several methodological limitations, such as selective attrition due to death or dropout, which can skew the results and prevent a clear understanding of the long-term effects of smoking cessation [5]. 

This study aims to examine the association between tobacco cessation and cognitive decline, with the central hypothesis that longer durations of cessation are associated with lower odds of cognitive decline. Cognitive decline refers to the gradual deterioration of mental abilities, including thinking, memory, and concentration, which are critical for daily functioning [6]. Previous research has suggested that smoking history can influence the progression of cognitive decline. For example, a study found that individuals with a history of smoking had a higher prevalence of mild cognitive decline and a more rapid reduction in entorhinal cortex volume [7]. The entorhinal cortex, a brain region involved in long-term memory formation, serves as the primary cortical input to the hippocampus and is crucial for processing facts and events [8,9]. This structural change in the brain may be a key factor in the cognitive impairments observed in smokers, underscoring the importance of examining how smoking cessation could mitigate these effects. 

A study observed that individuals who had quit smoking for at least 10 years did not show accelerated cognitive decline, in contrast to current smokers, who experienced faster cognitive deterioration compared to non-smokers [10]. Structural and functional brain imaging studies further support that longer cessation is associated with normalization of white matter integrity and brain network function [11,12]. This finding highlights the potential for recovery or stabilization of cognitive function in those who quit smoking long-term. The full scope of the cognitive benefits from smoking cessation remains unclear, particularly regarding how long it may take for these benefits to emerge and whether they are sufficient to counteract the cumulative damage caused by years of smoking. However, as our study is cross-sectional, we focus on associations rather than establishing causal or temporal effects. 

Given the increasing recognition of tobacco smoking as a modifiable risk factor for cognitive decline, public health initiatives promoting smoking cessation have become more critical. Understanding the long-term effects of quitting smoking on cognitive health is essential for shaping these interventions and guiding policy decisions aimed at reducing dementia risk. If smoking cessation can be shown to significantly reduce cognitive decline over time, it would reinforce the importance of early intervention and smoking cessation programs as part of a comprehensive strategy to improve brain health and prevent dementia in aging populations. By investigating these associations, this study seeks to determine whether longer durations of smoking cessation are linked to lower odds of cognitive decline, providing evidence to support early cessation and brain health strategies in aging populations. 

## Materials and methods

The study data were derived from the 2023 Behavioral Risk Factor Surveillance System (BRFSS), a compilation of health-related telephone surveys that collect information from participants who are 18 years or older. We selected data from states that included optional modules addressing cognitive decline and tobacco cessation. The states included are Florida, Maine, Rhode Island, Utah, Vermont, Wisconsin, Nevada, and Indiana. This study utilized publicly available, de-identified data and was therefore exempt from institutional review board approval and informed consent requirements.

Participants under 45 years old were excluded due to non-participation in the cognitive decline module. The analytic sample was further restricted to respondents from states that administered the cognitive decline module. Cases with missing data on key variables, including cognitive decline status, tobacco cessation status, or selected demographic covariates, were excluded using listwise deletion. The data were weighted per BRFSS methodological guidelines [13]. Frequencies and percentages were computed for various categorical demographic variables, including race, sex, income, age, and education. Cognitive decline was determined by a ‘yes’ answer to the question, “Have you experienced confusion or memory loss that is happening more often or is getting worse?” Length of tobacco cessation was determined by the following answer choices to the question “How long has it been since you last smoked a cigarette, even one or two puffs?”: “Within the past month (less than 1 month ago)”, “Within the past 3 months (1 month but less than 3 months ago)”, “ Within the past 6 months (3 months but less than 6 months ago)”, “Within the past year (6 months but less than 1 year)”, “Within the past 5 years (1 year but less than 5 years ago)”, “Within the past 10 years (5 years but less than 10 years ago)”, “10 years or more”, or “Never smoked regularly.” Data were retrieved from the CDC BRFSS portal and imported into IBM Corp. Released 2023. IBM SPSS Statistics for Windows, Version 29. Armonk, NY: IBM Corp. for preparation and analysis. Cases with missing information on key variables such as SCD, tobacco cessation status, or major demographic characteristics were excluded. Variables were reformatted into categorical measures as needed, and BRFSS-provided sampling weights were applied in all analyses to generate population-representative estimates. Analyses were conducted in IBM Corp. Released 2023. IBM SPSS Statistics for Windows, Version 29. Armonk, NY: IBM Corp., using complex survey procedures to account for weighting, although stratification and clustering variables were not explicitly incorporated. Demographic covariates were categorized according to BRFSS standard groupings, including age (45-54, 55-64, ≥65 years), sex (male, female), race/ethnicity, income categories, and education levels as defined in the survey.

Cognitive decline and tobacco cessation variables were analyzed as categorical measures based on predefined BRFSS response categories. The study sample was described using descriptive statistics: categorical variables were summarized with frequencies and percentages, while continuous variables were presented as means with standard deviations or as medians with ranges, depending on distribution. To examine whether tobacco cessation was independently associated with cognitive decline, bivariate analyses were performed, employing chi-square tests for categorical variables and independent t-tests for continuous variables. Predictors meeting a significance threshold of p<0.10 were entered into a multivariable logistic regression model using backward conditional selection. Covariates were selected a priori based on established literature. Multicollinearity among covariates was assessed using variance inflation factors (VIF), and all variables met acceptable thresholds. 

This approach produced an adjusted model assessing the relationship between tobacco cessation and cognitive decline, controlling for potential demographic confounders. Adjusted odds ratios (ORs) with corresponding 95% confidence intervals (CIs) were reported. In the final regression model, variables that remained significant at p<0.05 were retained in the final, best-fitting model and considered statistically significant. 

## Results

Demographic characteristics, self-reported interval since last smoked, and prevalence of CD in each category can be found in Table 1. The prevalence of CD is increased in individuals with any history of smoking. There was also a general positive trend of increased CD with increased interval since last smoking. Demographic factors such as race, education, income, age, and sex were independently associated with self-reports of CD. The risk of CD increased with age, with a prevalence of 50.96% among individuals aged 65 years or older. Females exhibited a higher prevalence compared to males at 55.64%, and among racial groups, Caucasians had the highest prevalence of CD at 70.20%. There was no clear trend across income levels; however, individuals earning between $55,000 and $100,000 were at the highest risk of developing CD. Similarly, there was no clear trend across education levels. 

**Table 1:** Participant characteristics across cognitive decline status (n=14,993,186), BRFSS 2023* *Weighted data; n: Total number of participants. All percentages are based on the total n = 14,993,186 participants. Significance represents chi-squared values derived from bivariate analysis, with statistical significance defined as a chi-square p-value < 0.001*.

Variable (total n = 14,993,186)	Cognitive Decline		p-value
	Yes n (11.10%)	No n (88.90%)	
Interval Since Last Smoked			<0.001
Never smoked regularly	5,952.46 (1.03)	50,106.63 (1.23)	
10 years or more	396,616.50 (68.49)	3,001,267.55 (73.47)	
5 years to < 10 years ago	69,325.58 (11.97)	408,231.42 (9.99)	
1 year to < 5 years ago	61,067.10 (10.54)	398,275.23 (9.75)	
6 months to < 1 year ago	20,984.29 (3.62)	83,363.40 (2.04)	
3 to < 6 months ago	9,249.17 (1.60)	61,601.80 (1.51)	
1 to < 3 months ago	7,424.21 (1.28)	36,445.65 (0.89)	
< 1 month ago	8,503.79 (1.47)	45,882.20 (1.12)	
Age			<0.001
45-54	361,079.92 (22.51)	3,468,678.14 (26.30)	
55-64	425,529.28 (26.53)	3,777,634.75 (28.64)	
65>	817,311.34 (50.96)	5,942,585.95 (45.06)	
Sex			<0.001
Female	899,381.06 (55.64)	6,919,769.49 (52.26)	
Male	717,111.23 (44.36)	6,321,790.33 (47.74)	
Race			<0.001
White, Non-Hispanic	1,085,637.73 (70.20)	9,533,068.05 (74.16)	
Black, non-Hispanic	103,768.64 (6.71)	1,040,465.80 (8.09)	
Other race only, Non-Hispanic	50,735.12 (3.28)	384,335.92 (2.99)	
Multiracial, Non-Hispanic	72,585.52 (4.69)	295,876.03 (2.30)	
Hispanic	233,799.35 (15.12)	1,601,066.65 (12.45)	
Income			<0.001
	175,327.74 (13.54)	500,881.21 (4.72)	
$15000 - $25,000	268,160.70 (20.70)	1,048,601.15 (9.89)	
$25,000-$35000	197,914.14 (15.28)	1,236,745.96 (11.66)	
$35,000-$50,000	167,510.39 (12.93)	1,442,339.75 (13.60)	
$50,000-$100,000	282,537.90 (21.81)	3,522,421.97 (33.21)	
$100,000-$200,000	149,946.09 (11.58)	2,194,838.20 (20.69)	
>$200,000	53,877.37 (4.16)	661,458.74 (6.24)	
Education			<0.001
Did not graduate high school	303,631.27 (18.87)	1,197,548.27 (9.09)	
Graduated high school	467,344.38 (29.04)	3,628,494.43 (27.54)	
Attended college/Technical school	531,027.48 (32.99)	4,281,555.83 (32.50)	
Graduated College/Technical school	307,468.08 (19.10)	4,068,160.81 (30.88)	

As shown in Table 2, bivariate chi-square analysis demonstrated significant associations between the interval since last smoked and all examined demographic variables, including age, sex, race, income, and education. The overall association was also statistically significant.

**Table 2:** Chi-square analysis of sociodemographic characteristics and interval since last smoked in relation to cognitive decline Chi-square values derived from bivariate analysis. Statistical significance is defined as p < 0.001*.

Variable	χ² Value	Degrees of Freedom (df)	p-value
Interval Since Last Smoked	11203.11	7	<0.001*
Age	21060.84	2	<0.001*
Sex	6600.98	1	<0.001*
Race	45200.51	4	<0.001*
Income	396718.90	6	<0.001*
Education	203448.76	3	<0.001*

Table 3 presents the results from the multivariable logistic regression and provides the risk of experiencing CD in individuals with a history of tobacco cessation when adjusting for various demographic and socioeconomic factors. The findings indicate that compared to individuals who have never smoked, individuals who quit smoking 10 or more years ago were 3.648 (95% CI 3.495, 3.807) times more likely to report CD. There is a general trend demonstrating greater CD with shorter durations since smoking cessation. Individuals who quit smoking 1 month to less than 3 months ago had the highest odds ratio for developing CD at 5.466 (95% CI 5.197, 5.748) as compared to the reference group when adjusted for other demographic variables. African Americans with a history of tobacco cessation were less likely to develop cognitive decline compared to Caucasians, with an odds ratio of 0.647 (95% CI 0.637, 0.657), whereas multiracial, non-Hispanic individuals were 1.169 (95% CI 1.147, 1.191) times more likely to develop cognitive decline. Education level also played a significant role, as individuals who graduated from high school exhibited the lowest risk of cognitive decline compared to those who did not graduate from high school. Males with a history of tobacco cessation were 0.877 (95% CI 0.871, 0.882) times as likely to develop cognitive decline compared to females. Household income was another significant factor, with individuals earning between $15,000 and $25,000 having a 0.617 (95% CI 0.609, 0.625) times risk of cognitive decline compared to those earning below $15,000, while those with incomes exceeding $200,000 were 0.259 (95% CI 0.254, 0.264) times as likely to develop cognitive decline. Age was inversely associated with the risk of cognitive decline; compared to individuals under 55 years old, those aged between 55 and 64 years were 0.783 (95% CI 0.775, 0.790) times more likely to report cognitive decline, and individuals aged 65 years and older were 0.767 (95% CI 0.760, 0.773) times more likely to report cognitive decline.

**Table 3:** Risk of cognitive decline among individuals with a history of tobacco cessation, adjusted for race, income, education, age, and sex *Statistically significant at p < 0.05

Step 1a		B	S.E.	Wald	df	Sig.	Exp(B)	95% C.I. for EXP(B)	
								Lower	Upper
Interval Since Last Smoked	Never smoked regularly	-	-	7877.187	7	<0.001*	-	-	-
	10 years or more	1.294	0.022	3534.215	1	<0.001 *	3.648	3.495	3.807
	5 years to < 10 years ago	1.544	0.022	4833.231	1	<0.001 *	4.682	4.482	4.890
	1 year to < 5 years ago	1.380	0.022	3843.874	1	<0.001 *	3.975	3.806	4.153
	6 months to < 1 year ago	1.545	0.024	4306.705	1	<0.001 *	4.689	4.478	4.911
	3 to < 6 months ago	0.975	0.027	1353.962	1	<0.001 *	2.652	2.518	2.793
	1 to < 3 months ago	1.699	0.026	4366.973	1	<0.001 *	5.466	5.197	5.748
	< 1 month ago	1.369	0.025	2891.051	1	<0.001 *	3.931	3.740	4.132
Race	White, Non-Hispanic	-	-	7274.832	4	<0.001 *	-	-	-
	Black, Non-Hispanic	-0.435	0.008	3231.092	1	<0.001 *	0.647	0.637	0.657
	Other race only, Non-Hispanic	-0.269	0.010	751.699	1	<0.001 *	0.764	0.749	0.779
	Multiracial, Non-Hispanic	0.156	0.010	263.615	1	<0.001 *	1.169	1.147	1.191
	Hispanic	-0.370	0.006	3578.166	1	<0.001 *	0.691	0.682	0.699
Education	Did not graduate high school	-	-	7210.035	3	<0.001 *	-	-	-
	Graduated high school	-0.443	0.005	6776.638	1	<0.001 *	0.642	0.635	0.649
	Attended college/ Technical school	-0.377	0.005	4750.866	1	<0.001 *	0.686	0.679	0.693
	Graduated College/Technical school	-0.424	0.006	4661.372	1	<0.001 *	0.654	0.646	0.662
Income		-	-	84494.486	6	<0.001 *	-	-	-
	$15000 - $25,000	-0.483	0.006	5783.253	1	<0.001 *	0.617	0.609	0.625
	$25,000-$35000	-0.579	0.006	7976.151	1	<0.001 *	0.560	0.553	0.567
	$35,000-$50,000	-1.050	0.007	24847.372	1	<0.001 *	0.350	0.345	0.355
	$50,000-$100,000	-1.316	0.006	47452.821	1	<0.001 *	0.268	0.265	0.271
	$100,000-$200,000	-1.782	0.007	57253.866	1	<0.001 *	0.168	0.166	0.171
	>$200,000	-1.352	0.010	18109.933	1	<0.001 *	0.259	0.254	0.264
Gender	Male	-0.132	0.003	1614.406	1	<0.001 *	0.877	0.871	0.882
Age (years)	45-54	-	-	3774.777	2	<0.001 *	-	-	-
	55-64	-0.245	0.005	2599.090	1	<0.001 *	0.783	0.775	0.790
	65>	-0.266	0.005	3410.790	1	<0.001 *	0.767	0.760	0.773
	Constant	-1.594	0.022	5202.697	1	<0.001 *	0.203	-	-

**Figure 1:**
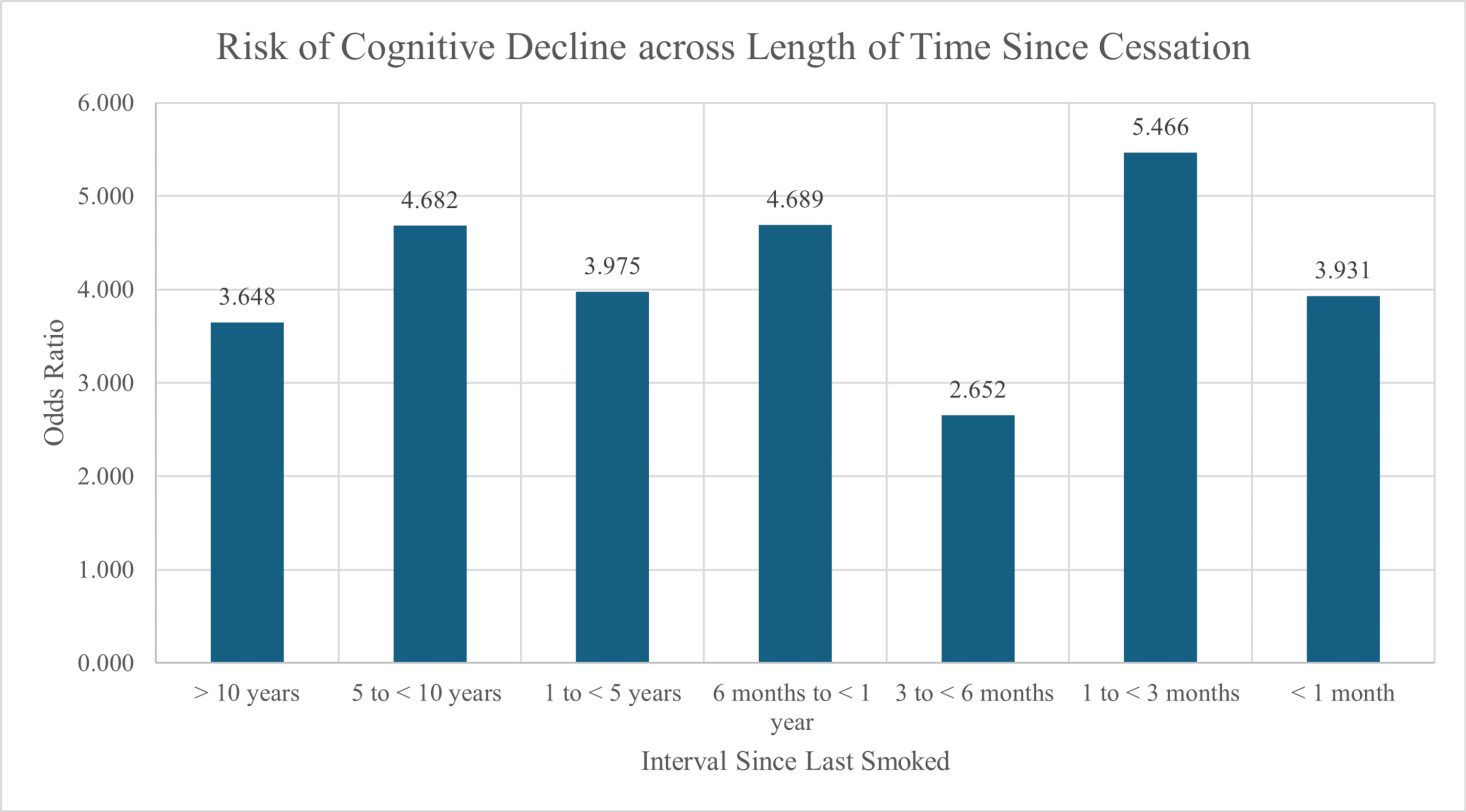
Inverse relationship between cognitive decline and tobacco cessation intervals

Figure 1 reinforces that the odds ratios reveal a distinct temporal gradient in CD risk following smoking cessation. Individuals with the shortest duration since quitting, particularly 1 to <3 months (OR = 5.466) and 6 months to <1 year (OR = 4.689) exhibited the highest odds of CD compared with never smokers. By contrast, participants who had abstained for longer periods, such as 3 to <6 months (OR = 2.652) or ≥10 years (OR = 3.648), demonstrated comparatively lower odds. These findings corroborate the regression analysis, indicating that shorter intervals since cessation are associated with elevated CD risk, whereas extended periods of abstinence confer a gradual reduction in risk, highlighting the long-term reduced CD risk of sustained smoking cessation.

## Discussion

This study investigated the association of length of time since tobacco cessation with cognitive decline in the United States of America among those above the age of 45. This data was obtained using BRFSS, a nationally representative state-based surveillance system of US adults. The study found that individuals who quit smoking 1 to less than 3 months ago were 5.466 (95% CI [5.197, 5.748]; p<0.001) times more likely to report cognitive decline compared to those who never smoked regularly, after adjusting for race, sex, income, and education (Table 3). In contrast, individuals who had quit smoking for at least 10 years were 3.648 (95% CI [3.495, 3.807]; p<0.001) times more likely to report cognitive decline compared to those who never smoked regularly when adjusted for the same variables (Table 3). These findings suggest that individuals who quit at least 10 years ago are less likely to experience cognitive decline compared to those who quit 1 to 3 months ago. Although this gradient suggests lower odds of cognitive decline with longer cessation duration relative to more recent quitters, the finding that even long-term quitters had higher odds than never smokers may reflect several factors. First, cumulative lifetime tobacco exposure may result in persistent vascular and neurologic damage that is not fully reversible despite prolonged abstinence. Second, reverse causation is possible, whereby individuals experiencing early cognitive symptoms may be more likely to stop smoking because of increasing health concerns. Finally, survivorship bias may contribute, as individuals with the most severe smoking-related morbidity or mortality may be less likely to survive or participate in survey-based analyses. A study found that individuals who had quit smoking for at least 10 years did not experience accelerated cognitive decline, while current smokers showed a faster rate of decline compared to those who had never smoked [10]. A prospective cohort study found that current smokers have the highest risk of cognitive decline and incident dementia, while recent quitters (<9 years) retain some elevated risk compared to never smokers [14]. However, those who quit ≥9-10 years prior show no significant excess risk for cognitive decline or dementia compared to never-smokers. This pattern is observed across diverse populations and cognitive domains, including global cognition, memory, and executive function. 

Additionally, males with a history of smoking cessation were 0.877 (95% CI [0.871, 0.882]; p<0.001) times as likely to report cognitive decline when compared to females, after adjusting for sex, income, and education (Table 3). This aligns with findings from a study that found that women who smoked in their early 60s were more likely to develop cognitive impairments when compared to men [15]. Another study found no significant effect modification by sex in the association between smoking status and subjective cognitive decline among middle-aged and older adults in the US, indicating that the relationship between cessation duration and cognitive decline is similar for men and women [16]. On the opposing end, a study found that cigarette smoking may contribute to cognitive impairment in men, as evidenced by lower MoCA scores and higher CSF levels of iron, zinc, lead, and aluminum, indicating an accelerated cognitive decline compared to women [17]. Therefore, the impact of smoking cessation on cognitive decline may differ between males and females, as evidenced by the lower MoCA scores and elevated levels of iron, zinc, lead, and aluminum in the CSF. These differences could influence how quickly cognitive decline progresses after quitting smoking, as men and women may experience distinct changes in vascular health, neuroinflammation, and brain function. Given that smoking affects brain health through its impact on cardiovascular and metabolic systems, the rate of cognitive decline following smoking cessation may differ between the sexes. Routine cognitive evaluations for both men and women are essential to identify early signs of cognitive impairment and intervene before a significant decline occurs. Understanding the sex-specific effects of smoking cessation on cognitive health is important for developing tailored public health strategies and personalized care plans. By addressing gender differences in how smoking cessation influences brain health, healthcare providers can better support individuals in maintaining cognitive function and slowing the progression of cognitive decline.

This study also finds that individuals who quit smoking and identify as multiracial, non-Hispanic were 1.169 (95% CI [1.147, 1.191]; p<0.001) times more likely to develop cognitive decline compared to Caucasians when adjusted for sex, income, and education (Table 3). There is limited research published on tobacco cessation in multiracial, non-Hispanic populations. A study investigating smoking cessation among racial/ethnic minorities found in their research that no intervention studies have focused on smokers from multiple racial backgrounds (e.g., biracial or multiracial individuals) or provided separate outcome data for this racial group [18]. Another study found that biracial and multiracial individuals exhibited higher smoking prevalence rates compared to monoracial or monoethnic groups amongst adolescents and young adults [19]. Reasoning for this may be due to the interaction between diverse family and social environments, and socioeconomic status may contribute to distinct pathways for both the initiation and cessation of smoking. As evidenced by the limited research on tobacco cessation and its effects on cognitive decline in multiracial populations, there is a pressing need for future research focused on this group to target more effective smoking cessation interventions to ultimately decrease the rate of cognitive decline in this population.

Our study found that as income increases, the risk of cognitive decline decreases when adjusted for sex, income, and education. Study participants who have quit smoking and earn an income of $15,000 - $25,000 were 0.617 (95% CI [0.609, 0.625]; p<0.001) times as likely to develop cognitive decline, whereas participants who quit smoking who earn greater than $200,000 were 0.259 (95% CI [0.254, 0.264]; p<0.001) times as likely to develop cognitive decline (Table 3). These results indicate an association between higher income and lower odds of cognitive decline among individuals who quit smoking. A study found that smoking is more prevalent among disadvantaged groups, who also face greater exposure to tobacco-related harms and have lower success rates in quitting, due to factors like reduced social support, stronger addiction, and limited access to effective treatments [20]. According to the study, to reduce smoking-related health inequalities, a combination of tobacco control measures, such as raising tobacco prices, targeted cessation programs, and mass media interventions, should be implemented alongside broader efforts to address social and health inequalities. 

This study has limitations inherent to the use of CDC BRFSS data. The use of self-reported data for both cognitive decline and smoking history introduces the potential for recall and reporting bias. Cognitive decline was assessed using a single subjective question, which may not accurately capture the severity or cause of impairment. Additionally, the cross-sectional design of the BRFSS prevents the establishment of causal relationships or temporal sequences between smoking cessation and cognitive decline. The analysis is further limited by the inclusion of data from only eight states that opted to administer both relevant modules, which may reduce the generalizability of the findings to the broader U.S. population. Participants under 45 years old were excluded, potentially omitting early-onset cognitive issues and smoking patterns relevant to younger adults. Residual confounding may also be present, as important variables such as comorbid conditions, mental health, and lifestyle factors may not have been fully captured or controlled for. Survivorship bias may be present since individuals with more severe cognitive impairment or adverse smoking-related outcomes may not have participated. Finally, the broad categorization of cessation timelines and the absence of detailed smoking history (e.g., pack-years) may limit the study’s ability to detect nuanced dose-response relationships between tobacco exposure and cognitive health. 

## Conclusions

This study concludes that individuals who have never smoked or who quit at least 10 years ago are less likely to experience cognitive decline, suggesting that smoking cessation is associated with a lower risk of cognitive deterioration. However, a limitation of this study is the reliance on self-reported data, which may introduce potential biases and affect the accuracy of the findings. Moreover, the brain-damaging effects of tobacco use may be reversed or halted if smoking cessation occurs, emphasizing the potential for cognitive health improvement post-cessation. 

Given these findings, early interventions such as routine cognitive screening and targeted smoking cessation counseling may play an important role in reducing the burden of cognitive decline. Future research should focus on longitudinal analyses to better assess temporal relationships and investigate the impact of cumulative smoking exposure and cessation timing on cognitive outcomes. 

## Appendices

**Table 4:** Module 13 - cognitive decline from 2023 BRFSS codebook report Variables derived from the Behavioral Risk Factor Surveillance System (BRFSS) 2023 Cognitive Decline [13].

Question Text	Variable Name	Responses
The next few questions ask about difficulties in thinking or remembering that can make a big difference in everyday activities. This does not refer to occasionally forgetting your keys or the name of someone you recently met, which is normal. This refers to confusion or memory loss that is happening more often or getting worse, such as forgetting how to do things you’ve always done or forgetting things that you would normally know. We want to know how these difficulties impact you. During the past 12 months, have you experienced difficulties with thinking or memory that are happening more often or are getting worse?	CIMEMLO1	1 - Yes
		2 - No
		7 - Don’t know/Not sure
		9 - Refused

**Table 5:** Module 15 - tobacco cessation from 2023 BRFSS codebook report Variables derived from the Behavioral Risk Factor Surveillance System (BRFSS) 2023 Tobacco Use Modules [13].

Question Text	Variable Name	Responses
How long has it been since you last smoked a cigarette, even one or two puffs?	LASTSMK2	1 - Within the past month (less than 1 month ago)
		2 - Within the past 3 months (1 month but less than 3 months ago)
		3 - Within the past 6 months (3 months but less than 6 months ago)
		4 - Within the past year (6 months but less than 1 year ago)
		5 - Within the past 5 years (1 year but less than 5 years ago)
		6 - Within the past 10 years (5 years but less than 10 years ago)
		7- 10 years or more
		8 - Never smoked regularly
		77 - Don’t know/Not sure
		99 - Refused
